# An *In Silico* study of TiO_2_ nanoparticles interaction with twenty standard amino acids in aqueous solution

**DOI:** 10.1038/srep37761

**Published:** 2016-11-24

**Authors:** Shengtang Liu, Xuan-Yu Meng, Jose Manuel Perez-Aguilar, Ruhong Zhou

**Affiliations:** 1 School for Radiological and Interdisciplinary Sciences (RAD-X) and Collaborative Innovation Center of Radiation Medicine of Jiangsu Higher Education Institutions, Soochow University, Suzhou, 215123, China; 2 Computational Biological Center, IBM Thomas J. Watson Research Center, Yorktown Heights, NY 10598, USA; 3 Department of Chemistry, Columbia University, New York, NY 10027, USA

## Abstract

Titanium dioxide (TiO_2_) is probably one of the most widely used nanomaterials, and its extensive exposure may result in potentially adverse biological effects. Yet, the underlying mechanisms of interaction involving TiO_2_ NPs and macromolecules, *e.g.*, proteins, are still not well understood. Here, we perform all-atom molecular dynamics simulations to investigate the interactions between TiO_2_ NPs and the twenty standard amino acids in aqueous solution exploiting a newly developed TiO_2_ force field. We found that charged amino acids play a dominant role during the process of binding to the TiO_2_ surface, with both basic and acidic residues overwhelmingly preferred over the non-charged counterparts. By calculating the Potential Mean Force, we showed that Arg is prone to direct binding onto the NP surface, while Lys needs to overcome a ~2 kT free energy barrier. On the other hand, acidic residues tend to form “water bridges” between their sidechains and TiO_2_ surface, thus displaying an indirect binding. Moreover, the overall preferred positions and configurations of different residues are highly dependent on properties of the first and second solvation water. These molecular insights learned from this work might help with a better understanding of the interactions between biomolecules and nanomaterials.

In nature, titanium dioxide (TiO_2_) exists in three polymorphic forms: rutile, anatase and brookite^1^. TiO_2_ nanomaterials have been extensively studied due to their unique physicochemical properties^2^ and, as early as 1970s, TiO_2_ was used as electrode for photoelectrolysis of water in electrochemical cell^3^. The photogeneration of a super amphiphilic TiO_2_ surface was then established, which displays excellent self-cleaning and antifogging features. Nowadays, TiO_2_-based nanomaterials have been used in paints, printing ink, paper, bio-medical ceramic and implanted biomaterials, and even as coloring agent in the food industry^4^,^5^,^6^. With the wide application of TiO_2_ in our daily life, there is also a growing concern on their potentially adverse health effects^7^,^8^,^9^,^10^,^11^,^12^ since the material is able to enter into the human body through direct skin contact, ingestion, and inhalation. Furthermore, interactions between macromolecules, such as proteins, and TiO_2_ surfaces are at the heart of many potential applications in bionanotechnology^13^. Therefore a better understanding of the nature of protein adsorption on TiO_2_ surfaces is of fundamental importance.

Molecular dynamics (MD) simulations have been widely used as a powerful and complementary tool to experiments, in the elucidation of the detailed interactions between biomolecules and nanomaterials^14^,^15^,^16^,^17^. Several recent simulation studies investigated the interactions of proteins and peptides with crystal TiO_2_ surfaces^18^,^19^,^20^,^21^. For instance, the experimental characterization of the peptide-titania interface has revealed that electrostatic interactions can play an important role in the binding process^22^. Despite these theoretical and experimental advances in the characterization of TiO_2_ surfaces, there is still little study on the interactions of amorphous TiO_2_ nanoparticles with standard amino acids, which is critical and necessary for a detailed understanding at atomic scale. Hence, in this study, we utilized atomistic MD simulations to characterize the energetic details involved in the interactions between the twenty standard alpha-amino acids and amorphous TiO_2_ NPs by exploiting our newly developed TiO_2_ force field^23^. We found that charged residues display stronger interactions relative to the rest of the amino acids suggesting a predominant role in the binding of proteins onto the TiO_2_ NPs.

## Models and Methods

The amorphous spherical TiO_2_ NP model with a radius of 17 Å was constructed. The Lennard-Jones (LJ) parameters for the TiO_2_ were taken from our recent work^23^. The values for the parameter ε, which described the potential well depth for the atom-atom interactions in the LJ potential, are as follows: ε = 2.4279 kJ/mol for the Ti-Ti interaction and ε = 1.2977 kJ/mol for the O-O interaction. Similarly, the values for the collision diameter σ are: σ = 0.196 nm and σ = 0.289 nm for the Ti-Ti and the O-O interactions, respectively. The cross interaction between the Ti and O atoms is determined by the Lorentz-Berthelot combination rule: 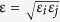, and 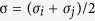. The atomic partial charge are 2.196 e and −1.098 e for the Ti and O atoms, respectively^24^.

To simplify the results and discussion, the twenty standard protein-forming alpha-amino acids are classified into four types: (i) Charged, consisting of the basic residues Arginine (Arg), Lysine (Lys), and the acidic residues Aspartic acid (Asp) and Glutamic acid (Glu); (ii) Aromatic, consisting of Phenylalanine (Phe), Tyrosine (Tyr) and Tryptophan (Trp); (iii) Polar, consisting of Asparagine (Asn), Serine (Ser), Glutamine (Gln), Threonine (Thr), Cystine (Cys) and Histidine (His); and (iv) Nonpolar, consisting of Glycine (Gly), Alanine (Ala), Proline (Pro), Leucine (Leu), Isoleucine (Ile), Valine (Val), and Methionine (Met).

**Figure 1:**
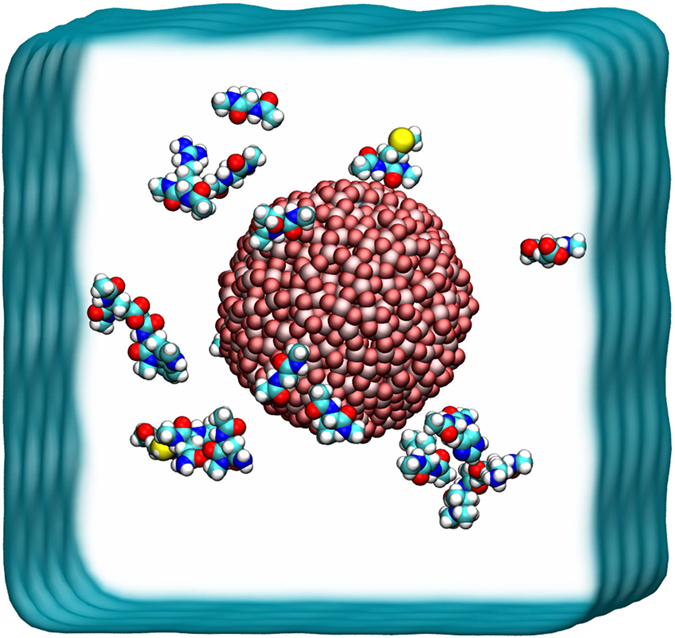
The initial configuration of the simulated system consisting of twenty alpha-amino acids and a single TiO_2_ NP in water solvated system. The dimensions of the water box are roughly 8 nm × 8 nm × 8 nm and it is rendered here as a blue surface. The positions of the twenty amino acids are randomly placed around the NP and are represented as vdW spheres. The titanium (Ti) and oxygen (O) atoms in the TiO_2_ NP are shown as pink and red van der Waals spheres, respectively.

To introduce a chemical environment into the individual amino acids that resembles that present in residues that are part of a protein, the N- and C-termini were capped with the acetyl (ACE) and N-methyl (CT3) groups, respectively. All the simulation systems were built and visualized using the VMD^25^ program (1.9.2 version). Each system consists of one spherical TiO_2_ NP and twenty capped amino acids embedded in a cubic water box of 8 nm in length (Fig. 1). The simulations were performed using the GROMACS (version 4.6.7) package^26^ program with the CHARMM22 force field^27^,^28^. The TIP3P water model^29^ was applied for water molecules. Prior to production runs, all systems were minimized and equilibrated with the protocol used in our previous studies^30^,^31^,^32^,^33^,^34^,^35^,^36^,^37^. During the equilibration and production runs, the temperature and pressure were maintained at 300 K and at 1 atm using a Langevin thermostat and barostat, respectively. The long-range Coulomb interactions were treated with the PME method^38^, while the van de Waals (vdW) interactions were handled with a smooth cutoff with a distance value of 1 nm. All the ten production runs were carried out for 100 ns each, resulting with a total aggregate simulation time of 1 μs, under the NPT ensemble. The equation of motion was integrated with a time step of 2 fs and coordinates were collected every 2 ps.

The adsorption of water and key amino acids on TiO_2_ NP surface was further investigated by estimating the free energy profiles through the calculation of the potential of mean constraint force (PMF). Umbrella sampling, which has been extensively used and is considered the gold standard free energy calculation method, was selected to estimate the free energy landscape associated with moving the different alpha-amino acids toward the NP. This technique was considered adequate for our purposes since there are no ‘slow structural responses’ along the defined pathway. Slow structural responses, such as those observed in the interactions in solute-membrane systems, exemplify cases where recent advanced methods provide a better description of the system^39^. The PMF was calculated along a reaction coordinate defined by pulling the capped amino acids toward the NP surface with a harmonic force of 2000 kJ mol^−1^nm^−2^ applied on the heavy atoms of the side chain to bringing it from a distance of 2.6 nm to 1.8 nm (the radius of the NP was 1.7 nm); in some cases a larger harmonic force of 5000 kJ mol^−1^nm^−2^ was applied on the heavy atoms at NP surface (1.8–2.0 nm) in order to improve samplings. The reaction coordinate was split into 9–11 windows with a length 20 ns for each run, thus amounting a total simulation of ~180–220 ns. Finally, the Weighted Histogram Analysis Method (WHAM)^40^,^41^ is applied to calculate the free energy; the statistical uncertainty of the PMF was estimated by bootstrapping analysis^42^ with an equilibration phase of 1 ns in length.

## Results and Discussions

The TiO_2_-water interface has been investigated using several experimental techniques (*e.g.,* X-ray crystallographic studies)^43^, as well as computational approaches (*e.g.,* molecular dynamics simulations)^44^,^45^,^46^. The results from these studies have showed that the surface of the TiO_2_ displays a significant hydrophilic character in aqueous solution. Moreover, using atomistic simulations, Skelton *et al*.^18^, highlighted the importance of structured water layers at the water-titania interface during the binding of a short oligopeptide. This prompted us to first investigate the role of water molecules at the proximity of amorphous TiO_2_ and their possible influence in regulating the absorption of the alpha-amino acids.

### Water behavior on the TiO_2_ NP surface

**Figure 2:**
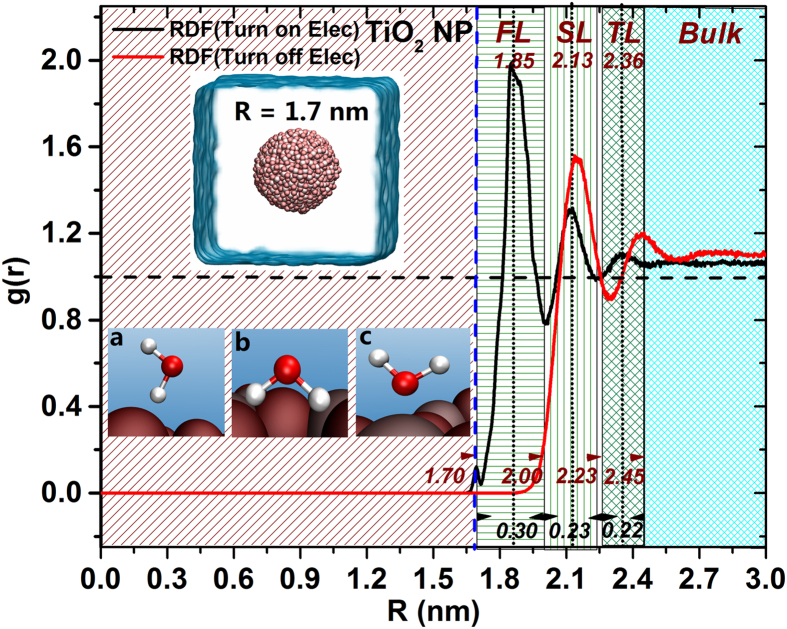
Water radial distribution function (RDF) profile and water molecular patterns during the adsorption events on the TiO_2_ NP surface with different water layers indicated in the background. The radius of the TiO_2_ NP is 1.7 nm (indicated here by a dashed blue line). The upper left inset displays the hydrated NP system, while representative water conformations are depicted on the lower left panel (a to c).

First we investigate the behavior and organization of water molecules at the surface of the TiO_2_ NP. We define the NP center of mass (COM) as the origin, unless otherwise specified, and the distance values related with the free energy calculations are presented in this framework. To determine the effect of the NP charge on the interfacial water layers, we turn on and turn off the NP electrostatic interactions. After 10 ns of MD production run, the radial distribution functions (RDF) profile for liquid water at the NP surface was calculated and shown in Fig. 2. Clearly, by turning on the electrostatic interactions (on-state), three distinctive water layers with different thickness were identified at the NP interface: a first layer (FL) that peaks at 1.85 nm with a thickness of 0.30 nm, a second layer (SL) that peaks at 2.13 nm with a thickness of 0.23 nm, and a third layer (TL) that peaks at 2.36 nm with a thickness of 0.22 nm. In contrast, by tuning off the electrostatic interaction (off-state), only two water layers were identified, the first one at 2.14 nm and the second at 2.44 nm. These results indicate that NP-water electrostatic interactions lead to the population of a dense first water layer (g(r) = 1.99), which peaks at distance from the origin of 1.85 nm. The second water layer for the on-state case, which peaks at a distance of 2.13 nm, is located a little bit closer to the first water layer than in the case of the off-state. This is ascribed to the fact that the external oxygen atoms protrude further in the NP surface than the Ti atoms, in such a way that the oxygen atoms can attract water molecules by forming stable hydrogen bonds (see lower left inset a, b shown in Fig. 2, define as O-Water). Additionally, the strong partial electrostatic interactions of the titanium atoms impel the adsorption of water molecules onto the NP surface by establishing interactions with the oxygen atoms from water and its forming pattern is displayed in inset c in Fig. 2 (define as Ti-Water). The formation of the Ti-Water interactions are responsible for the concentration of the water molecules that constitute the dense water layer (FL) observed in the on-state. To further investigate the water adsorption behavior at the NP surface, we calculated the hydrogen bond lifetime for the water molecules within the first hydration layer from the NP surface (Fig. S1). We found that the hydrogen bond lifetime become longer when the electrostatic contributions are turned on (hb life is 1.79 ps for the turn off-state, and 2.53 ps for the on-state, within 0.3 nm shell of NP surface, respectively). Longer hydrogen lifetimes imply that water molecules can reside on NP surface for longer time and aid in mediating solute adsorption on the NP surface. In this regard, Skelton *et al*.^18^, have indicated that the peptide (RKLPDA) may initially recognize the first two water layers at the rutile TiO_2_ (110) interface (not in the case of the titania surface), albeit no detailed mechanisms were investigated. Below we address this mechanistic void by calculating the adsorption free energy profile for different alpha-amino acids.

### Adsorption probability for single alpha-amino acids on the TiO_2_ NP surface

Since single alpha-amino acids constitute the basic building units for larger biomolecular entities, such as peptides or proteins, a detailed investigation of their interactions with material’s surfaces is essential for understanding and elucidating the behavior of protein adsorption events on nanomaterial interfaces. In these regards, recent computational studies investigated the adsorption of amino acids, short peptides, and amino acid analogues onto the TiO_2_ surface using umbrella sampling and metadynamics methods to calculate the PMF^20^,^21^. The study provided estimated free energy of adsorption of amino acid analogues on rutile TiO_2_ (110) surface. Also, Monti and co-workers^47^ have used temperature accelerated dynamics in tandem with the parallel-replica (PR) method, to estimate the binding free energy for the tripeptide H-KEK-NH_2_, on a range of different inorganic substrates, including the titania rutile (110) surface. Moreover, using classical reactive (ReaxFF) and nonreactive molecular dynamics simulations, Cui Li *et al*.^19^, have indicated that glycine had favorable binding free energy values and stable contacts on the TiO_2_ (110) surface, which were mainly mediated by its backbone carboxyl group. These pioneering investigations found that the amino (+NH_3_) and carboxyl (COO-) termini of the studied amino acids, were able to interact with the surface of titania and hence, these interactions are important in the amino acids binding mechanism. As a consequence, the transferability and generality of the amino acids-surface findings from these studies for cases where the amino acid residues are part of a larger system *i.e.*, peptides or proteins, become less reliable since only the first and last amino acid residues contain the backbone charged terminal groups. To address this limitation, here we included the acetyl (ACE) and N-methyl (CT3) capping groups at the N- and C-terminus in every amino acid, respectively. The twenty alpha-amino acids are classified into four groups – Charged, Aromatic, Polar, and Nonpolar – depending on general chemical properties of their side chains (see the “Model and Methods” sections for details).

**Figure 3:**
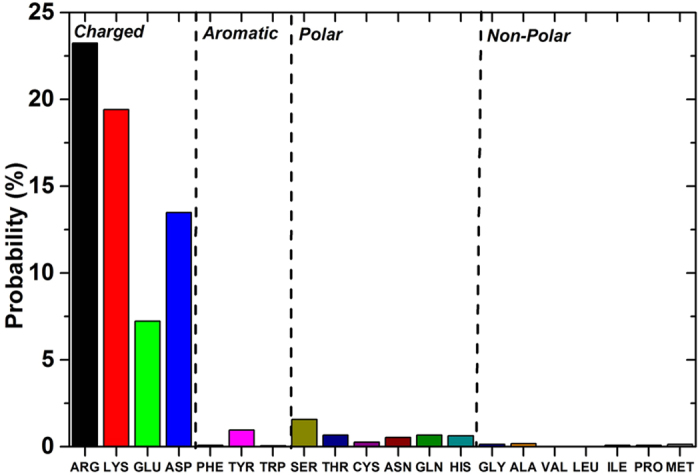
The total adsorption probability of the twenty alpha amino acids onto the TiO_2_ NP surface. Here, adsorption is counted when any side chain heavy atom is within 5 Å of the TiO_2_ surface. The four groups of amino acids – Charged, Aromatic, Polar, and Nonpolar – are separated by a dash line.

Figure 3 shows the total adsorption probability for each amino acid, which was calculated by integrating the probability distribution over a distance range of 0–5 Å (*i.e.*, counted when any side chain heavy atom is within 5 Å of the TiO_2_ surface). Notably, we found that the adsorption probabilities for the charged residues – arginine (Arg), lysine (Lys), aspartate (Asp), and glutamate (Glu) – are significantly higher than the probabilities for the other amino acid groups (aromatic, polar, and nonpolar; see the “Model and Methods” sections for details). In the charged group, the basic residues, Arg and Lys, show the highest adsorption probabilities with values of 23.2% and 19.4%, respectively, followed by the two acidic residues, Asp (13.5%) and Glu (7.2%), which have relatively lower adsorption probabilities. At the other extreme, all the non-charged amino acids show very weak adsorption probability values to the TiO_2_ NP. Also we found that residues with a hydroxyl group in their side chains, *e.g.*, the aromatic Tyr and the polar Ser and Thr, display slight preference for positioning in the first water layer, which is consistent with previous results^20^, where the methanol molecule was used as a surrogate for Ser (Fig. S2). Additionally, residues with the carboxamide group in their side chain, that is polar residues asparagine (Asn) and glutamine (Gln), can be adsorbed either in the first water layer or in the NP surface (Fig. S2). Consistent with our findings, a number of recent studies^48^,^49^,^50^ has suggested the pivotal role of charged and polar residues to promote the adhesion of short oligopeptide onto the surface of titania. In any case, amino acids from the charged group dominate the adsorption events. Therefore, in the following sections we will focus on elucidating the detailed mechanisms involved in the adsorption of Arg, Lys, Asp, and Glu on the TiO_2_ NP surface.

### Interaction between charged amino acids and TiO_2_ NP surface

The unique structure and (partial) charge distribution of the TiO_2_ NP cause the differential adsorption features presented by the alpha-amino acids. On the TiO_2_ NP surface, the ratio of Ti and O atoms is 1:2 and each Ti atom coordinates with three or four O atoms (see in Fig. 1). Thus the O atoms (−1.098 e) are more exposed than the Ti atoms on the TiO_2_ surface, which facilitates the preferred adsorption of basic residues and somehow inhibits that of the acidic ones. To better understand the differences in these adsorption patterns, we need to examine the details of the interactions between the TiO_2_ NP and the side chains of Arg, Lys, Glu, and Asp, as well as the role of the first two water layer during the adsorption.

### Interaction between basic residues and TiO_2_ NP surface

Arginine and lysine are similar in structure: the sidechain of Arg consists of a 3-carbon aliphatic chain followed by a guanidinium group, while Lys has a (-CH_2_)_4_ aliphatic chain capped by an amino group (-NH_3_^+^). The positive charge of Arg is delocalized on the guanidinium group enabling the formation of multiple H-bonds (or electrostatic interaction “centers”). The ε-amino group of Lys can also participate in the formation of multiple hydrogen bonds, albeit less prominently than the Arg guanidinium group^51^.

**Figure 4:**
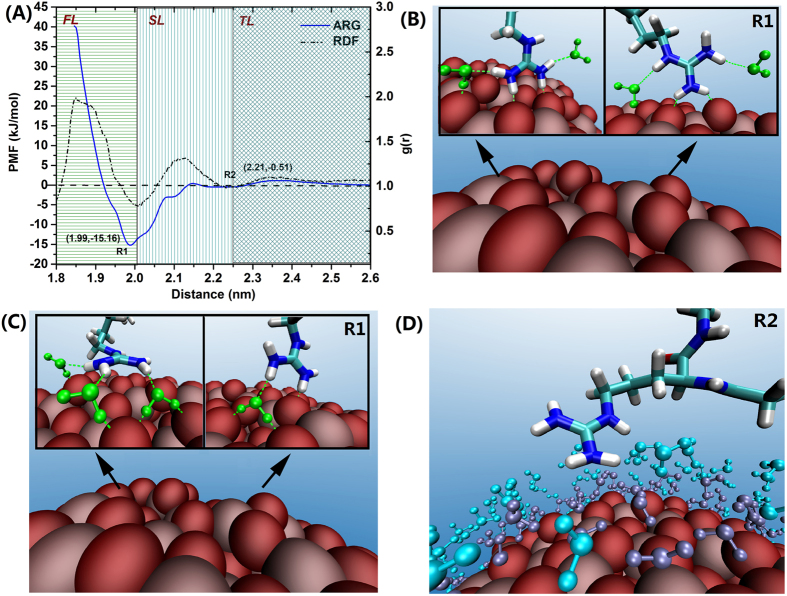
Free energy profile of the adsorption of Arginine on the TiO_2_ NP surface and representative configurations (**A**–**D**). The H-Bonds in the triad water-amino acid-TiO_2_ NP, are indicated by green dash lines. (**D**) Water molecules in the first (FL) and second (SL) water layers are colored in purple and cyan, respectively.

As shown in Fig. 4A, the free energy profile of the Arg adsorption at the NP interface features two minima, marked as R1 and R2, at a distance of 1.99, 2.21 nm, respectively. At R1, the guanidinium group of Arg directly forms one or two hydrogen bonds with O atoms in the TiO_2_ NP surface, forming stable adsorbed conformations as shown in Fig. 4B. Moving from bulk solvation to R2 and then to R1 actually delineates the processes of Arg’s diffusion, gradual contact and adsorption. Yet, few studies mentioned the waters’ role on these process. Here we found that, interestingly, the Arg free energy outline coincides with the water RDF profile, where the location of its deepest minimum closely overlaps with the interface between the first (FL) and second (SL) water layers (2.00 nm), with a value of −15.16 kJ/mol (R1); and R2, is located at the interface between the second (SL) and third (TL) water layer (2.23 nm) with an energy value of 0.51 kJ/mol. Arg at R1 forms hydrogen bonds with FL’s waters in addition to Arg-TiO_2_ H-bonds, which further stabilizing its conformation (Fig. 4B,C). Also at R2, Arg hydrogen bonds with SL’s waters (Fig. 4D). Thus waters in the FL and SL cooperatively involve in the Arg’s adsorption on TiO_2_ NP.

From the view of energy, the PMF curve indicates that, almost no energy barrier needs to overcome when Arg moves from R2 to R1, which implies Arg can freely adsorb on the NP surface without any free energy cost. This is because the guanidinium group can equally displace water molecules from the first water layer and form H-bonds with both TiO_2_ and surrounded waters, thereby offset the entropy decrease associated with the organization of water molecules in the proximity of the TiO_2_ NP surface (first water layer). In the other words, Arg is capable of placing its side chain in the first water layer without the need to drain off water molecules from this region, due to its ability of adopting stable conformations by forming an extensive hydrogen bonding network.

On the other hand, moving Arg from the NP surface toward the bulk (desorption process) requires large free energy ~15 kJ/mol; it needs to overcome not only the attractive electrostatic interactions established with the NP, but also the entropy decrease associated with the ordering of water molecules on the NP surface. Overall, the adsorption of Arg involves its direct electrostatic attraction to O atoms on the TiO_2_ surface as well as hydrogen bonding with surface waters; the compact first water layer does not play negative roles during the adsorption process.

**Figure 5:**
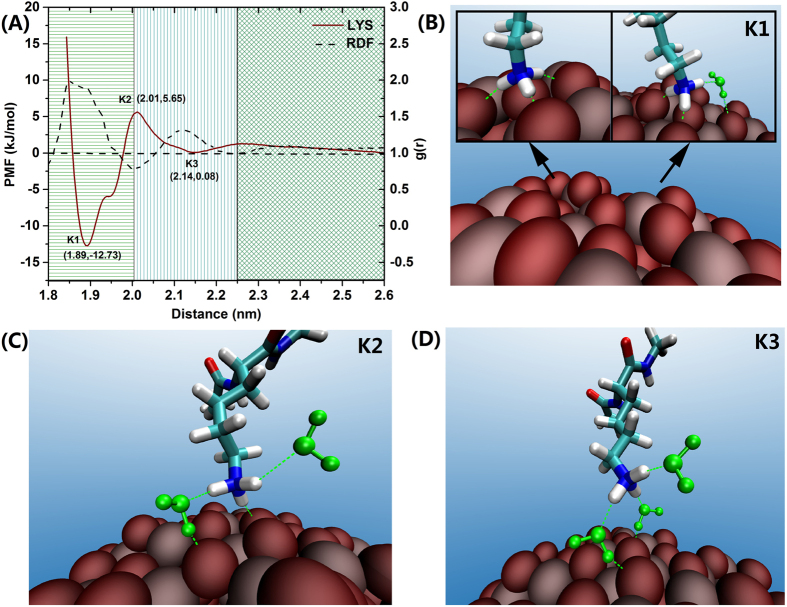
Free energy profile of the adsorption Lys on the TiO_2_ NP surface and representative configurations (**B**–**D**). The H-Bonds in the triad water-amino acid-TiO_2_ NP, are indicated by green dash lines.

In the case of Lys, the adsorption free energy contains two minima, K1 and K3, at 1.89 nm and 2.14 nm, with an associated energy of −12.73 kJ/mol and 0.08 kJ/mol, respectively. There is a free energy barrier between the K1 and K3 minima (moving from the bulk toward the surface) of 5.65 kJ/mol (~2 kT at room temperature) at a distance separation from the surface of 2.01 nm (K2), as indicated in Fig. 5A. The difference between the energy profiles for both basic residues can be ascribed to the distinct properties of the guanidinium and amino side chain groups. Unlike the terminal group in the Arg side chain, which displays a relatively large, rigid, and planar geometry, the terminal group in Lys is smaller in size and less capable of establishing a large number of electrostatic interactions (smaller hydrogen-binding capability). Thus, and in contrast to Arg, during the Lys absorption onto the NP surface, only a maximum of three hydrogen bonds can be formed with O atoms from the TiO_2_ NP, which would not be enough to offset the reduction in entropy related with the organization of water molecules in the first water layer, resulting in the presence of an energy barrier in the Lys free energy profile (Fig. 5A and B). The second minima, K3, (Fig. 5D) in the Lys adsorption profile is located at a distance from the surface (2.14 nm) that corresponds to the second water layer region (SL). The small value for this energy well (0.08 kJ/mol) indicates an almost free movement from the SL toward the bulk water region. Similarly to the case of Arg, the water molecules from the first water layer can establish favorable hydrogen bond interactions with the amino side chain group of Lys to stabilize its absorption onto the NP surface. In sharp contrast, K2 is in transition point and water molecules from the second water layer favor the desorption of Lys on the NP surface, as shown in Fig. 5C.

### Interaction between acidic residues and TiO_2_ NP surface

Relative to the case of the basic residues, the acidic residues display a somehow more complicated mechanism when they are adsorbed onto the TiO_2_ NP surface. The difference may be attributed to the negatively charged terminal side chain in the acidic residues and the presence of O atoms in the TiO_2_ NP, which are not only extensively located in the surface but contain as well a significant negative charge that could prevent direct adsorption. Nevertheless, some Ti atoms, with a charge of 2.196 e, are still exposed in the surface (despite the large presence of surface O atoms), which can interact with the terminal O atoms from the carboxylate groups (-COO^−^) of the acidic residues Glu and Asp.

**Figure 6:**
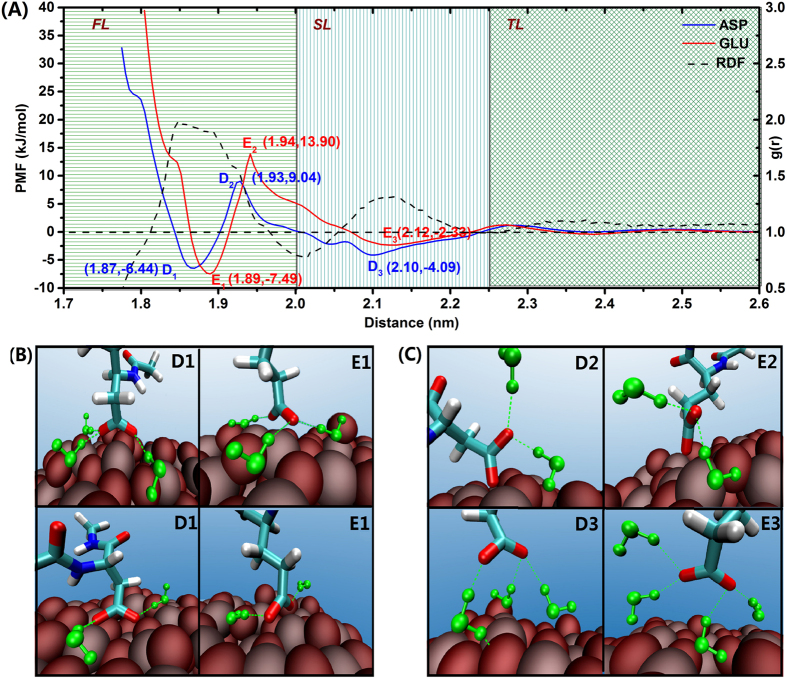
Free energy profile of the adsorption the acidic residues Asp and Glu on the TiO_2_ NP surface and representative configurations (**B**,**C**). The H-Bonds in the triad water-amino acid-TiO_2_ NP, are indicated by green dash lines.

The adsorption free energy profiles for both acidic residues are qualitatively similar and, when compare with the water RDF profile, particularly symmetric but opposite features are evident (Fig. 6A). The Asp profile displays two minima at 1.87 nm (D1) and 2.10 nm (D3) with an in-between maximum at 1.93 nm (D2); in the case of Glu, the corresponding values are: 1.89 nm for E1, 2.12 nm for E3, and 1.94 nm for E2.

The Asp and Glu exhibit similar chemical and structural properties and display generally similar free energy profiles. Their adopted configurations on the NP surface are also similar, as shown in Fig. 6B,C, where the terminal O atoms from the acidic side chain are able to interact with either one or two Ti atoms. Such adsorption configurations of Glu and Asp have been also deduced from infrared spectroscopy and vibrational spectroscopy observations^52^,^53^.

Despite of the similarity, the difference is also obvious between the acid residues: the Glu energy profile shows not only the position of the global minimum shifted toward larger distance values but also a higher energy barrier when compare with the Asp profile (Fig. 6A). We attribute this difference to the longer hydrophobic side chain of Glu, which contains an additional methylene group (-CH_2_) that makes the Glu side chain softer than the Asp side chain, and also to the larger conformational flexibility of the Glu. These factors limit the configurational adaptability of Glu to fit into a unique charge distribution in the proximity of the TiO_2_ NP surface, which leads to its relatively lower adsorption capability when compared with Asp, as indicated in Fig. 3.

It is interesting to make a comparison between basic and acid residues on their adsorption behavior. Energetically, when the acidic residues move toward the NP surface, that is, from D3 (E3) to D1 (E1), they need to overcome an energy barrier of 13.13 (17.99) kJ/mol, which is significantly higher than those found for the basic residue cases (0 and 5.6 kJ/mol for Arg and Lys, respectively). This means that the acidic residues not only need to conquer the protrusion of the O atoms present in the TiO_2_ NP surface but also the entropic-related inhibitory effect from the first and second water layers.

Once the acidic residues fall into the deepest free energy well in the free energy profile (D1/E1), there are at least two or three water molecules bound to the terminal carboxylate group via hydrogen bonds (Fig. 6B). In this context, water molecules from the second layer (SL) are prone to drag the side chains out from first water layer (Fig. 6C). Additionally, our calculations indicated that acidic residues are stably placed in the second minima (D3/E3), where are largely stabilized by interaction with SL water molecules (Fig. 6C); similar “indirect” binding configuration on the surface has been described previously^18^.

In terms of the water layer patterns in the charged amino acids, we observed interesting differences between the basic and acidic residues, that is, in order to establish interaction with Ti atoms, the carboxylate groups in the side chain of the acidic residues need to displace water molecules (FL) that are interacting with Ti atoms (associated binding free energy is −10.69 kJ/mol, see in Fig. S3), albeit without significant contributions from the water molecules (FL) interacting with O atoms (associated binding free energy is −0.55 kJ/mol, see in Fig. S3). In other words, and different to the basic amino acids, the side chains from the acidic groups need to displace water molecules from the first layer, which will cost a high energy relative to the case of the positively charged amino acids.

Moreover, the large difference between the energy values associated with the direct adsorption of the charged residues onto the NP, where the binding interactions of the basic residues are stronger than those of the acidic residues (R1/K1 versus D1/E1), might also contribute significantly to their differential adsorption behavior.

To further illustrate the adsorption mechanism, we also turned off the electrostatic interactions between TiO_2_ NP and the charged amino acids by setting TiO_2_ NP atomic partial charge to zero and calculated the respective free energy profiles. The results summarized in the Fig. S4. Lost the electrostatic attraction the Lys, Glu and Asp no longer adsorbed on the TiO_2_ surface as expected. What surprised us is, Arg still have free energy well (around −11.0 kJ/mol at ~2.12 nm) though a little bit shallow and right shifted compared to the electrostatic-on state (−15.16 kJ/mol at 1.99 nm, Fig. 4A), which means Arg is able to adsorb on the TiO_2_ surface even without electrostatic interaction. We speculated that the planar guanidinium of Arg may form strong vdW interaction with TiO_2_ and lead to the stable adsorption. Thus classical MD simulations were set up to test whether Arg, Phe and Asn, which have planar moiety, can be adsorbed on the ‘no-charge’ TiO_2_ NP. All of these residues indeed show adsorption. Moreover, a PMF calculation on the Phe also confirm a ~10 kJ/mol free energy well on the ‘no-charge’ TiO_2_ (see Fig. S5). Both Phe and Arg use their planar moiety, benzene and guanidinium, respectively, to stably pack onto the TiO_2_ surface. Additionally, as we discussed in the Fig. 1A, turn-off the TiO_2_ charges causes the loss of the first water layer, which may be another advantage for the adsorption of Phe considering the hydrophobic interaction. It also somehow reflects the important role of the water layers. Noted that a ‘no-charge’ TiO_2_ NP is not real situation. Yet it still help us to deeply understand the molecular mechanism of the amino acids’ adsorption as well as the water’s role during the adsorption process.

## Conclusion

In this study, we investigated the interactions of all twenty alpha amino acids with a spherical TiO_2_ nanoparticle using molecular dynamics simulation and pulling simulation methods. We found that charged amino acid residues – Arg, Lys, Glu, and Asp – are overwhelmingly more prone to be adsorbed onto the TiO_2_ NP surface with significantly stronger binding affinity than those non-charged amino acids, which is in good agreement with recent experimental findings^49^,^53^,^54^,^55^ and previous simulations on simplified amino acid analogues on the rutile titania (110) surface^20^,^21^. Within the charged group, the basic residues (Arg and Lys) are favored over the acidic residues (Glu and Asp) when adsorbing onto the TiO_2_ NP due to the dominant number of Oxygen atoms at the TiO_2_ surface. Arg is favored to be direct adsorbed onto NP surface without any free energy barrier, while Lys has to conquer about 2 kT energy barrier. Relative to the direct interaction of the basic residues with the TiO_2_ surface, the acidic residues are preferred to be indirectly bound onto the TiO_2_ surface (Ti atoms) by establishing interactions with water molecules localized in the first water layer.

Our current results compare favorably with available experimental data and provide significantly more atomic-level details that help to better understand the molecular mechanism of the adsorption for the most basic protein building blocks on the highly relevant TiO_2_ NP surface. Such understanding will be useful in the development of rational principles for the design of peptide sequences with predictable and controllable binding properties for TiO_2_ nanomaterials.

## Additional Information

**How to cite this article**: Liu, S. *et al*. An *In Silico* study of TiO_2_ nanoparticles interaction with twenty standard amino acids in aqueous solution. *Sci. Rep.* **6**, 37761; doi: 10.1038/srep37761 (2016).

**Publisher’s note:** Springer Nature remains neutral with regard to jurisdictional claims in published maps and institutional affiliations.

## Supplementary Material

Supplementary Information
