# Supplementary material for: An In Silico study of TiO2 nanoparticles interaction with twenty standard amino acids in aqueous solution
Source: Sci Rep. 2016 Nov 24;6:37761. doi: 10.1038/srep37761 (PMC5121885; doi:10.1038/srep37761)
Supplement: Supplementary Information [file srep37761-s1.pdf]

Supporting Information

An *In Silico* study of TiO_2 nanoparticles interaction with twenty standard amino acids in aqueous solution

Shengtang Liu[1‡], Xuan-Yu Meng[1‡], Jose M. Perez-Aguilar[2], and Ruhong Zhou[2,3,*]

1. School for Radiological and Interdisciplinary Sciences (RAD-X) and Collaborative Innovation Center of Radiation Medicine of Jiangsu Higher Education Institutions, Soochow University, Suzhou, China, 215123

2. Computational Biological Center, IBM Thomas J. Watson Research Center, Yorktown Heights, NY 10598, USA

3. Department of Chemistry, Columbia University, New York, NY 10027, USA

‡These authors contribute equally

* To whom correspondence should be addressed. E-mail: ruhongz@us.ibm.com (R. H. Z.)

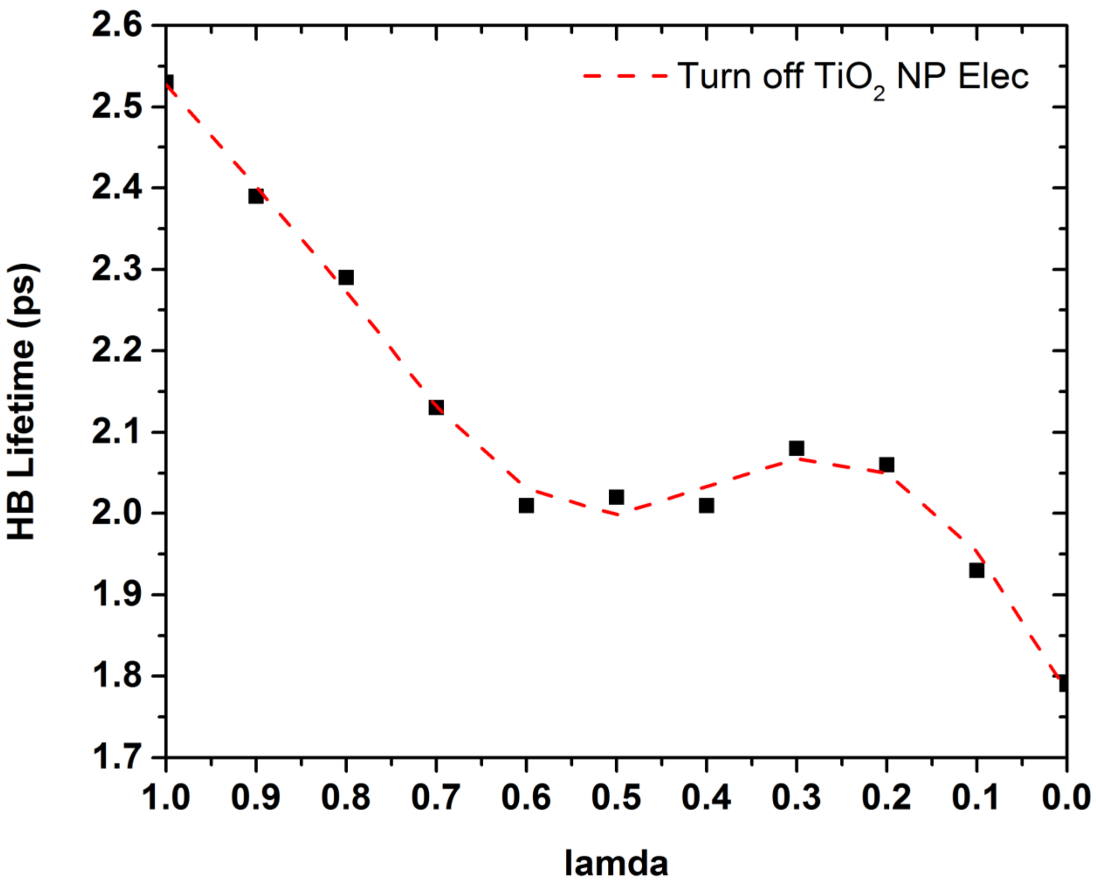

Fig. S1. Hydrogen bond lifetime profile when turning off TiO_2 NP atomic partial charges. The lamda is coefficient applied to TiO_2 atomic charge, lamda=1 stands for turning on atomic charge totally, lamda=0 means completely turning off the charges.

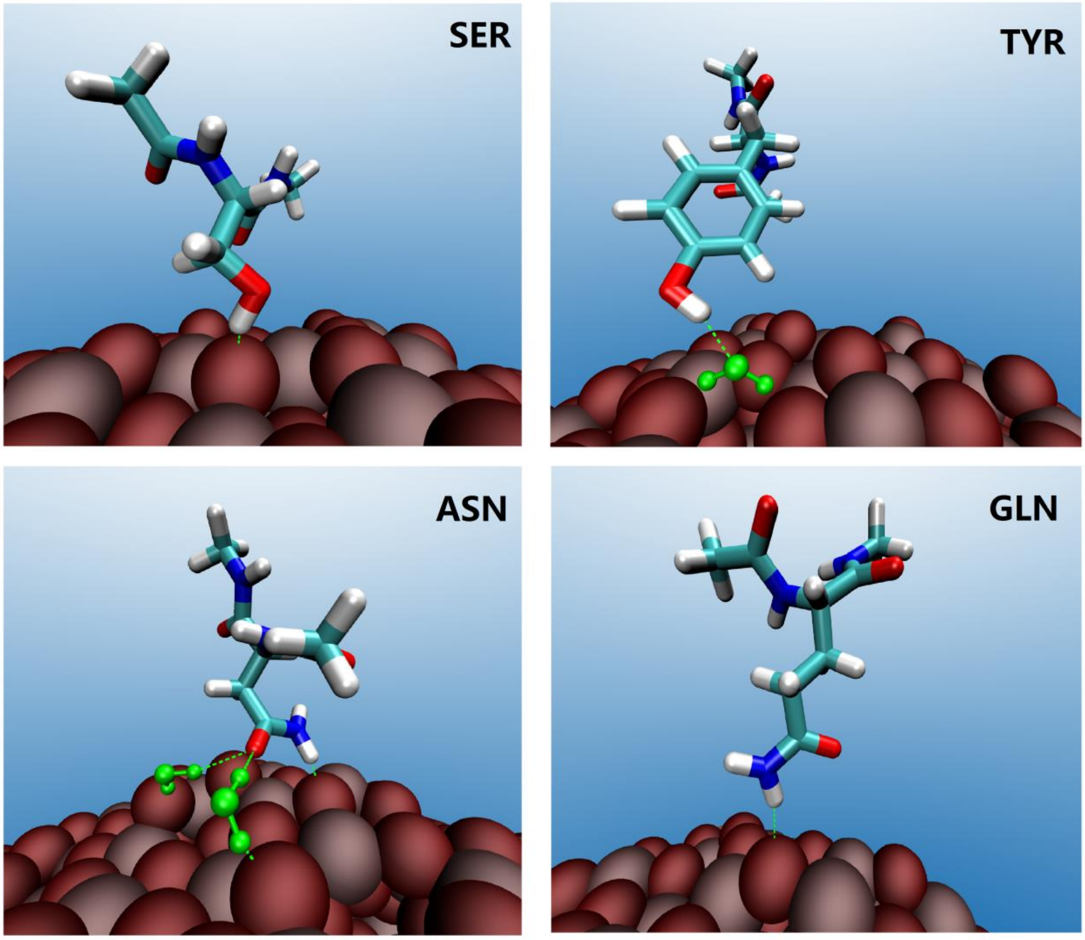

Fig. S2. Representative configuration of Ser, Tyr, Asn, Gln adsorbed onto the TiO_2 nanoparticle surface.

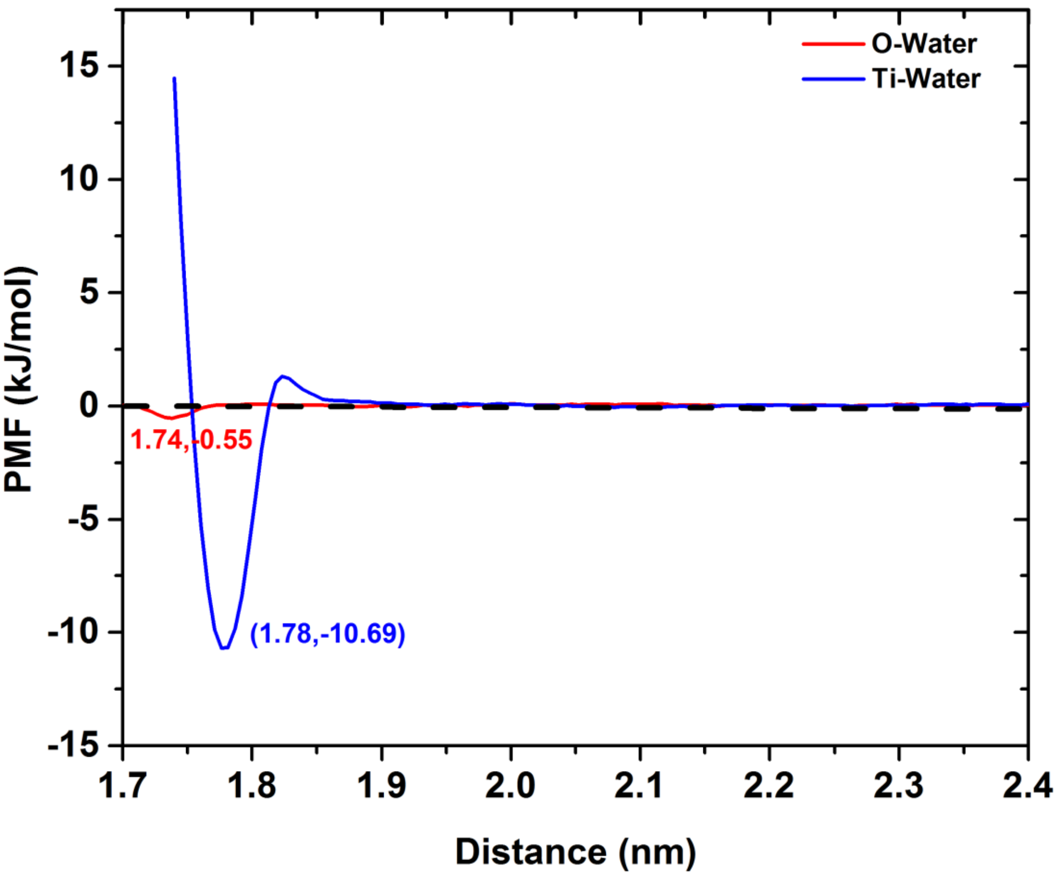

Fig. S3. Free energy profile of the adsorption of Ti-Water and O-Water.

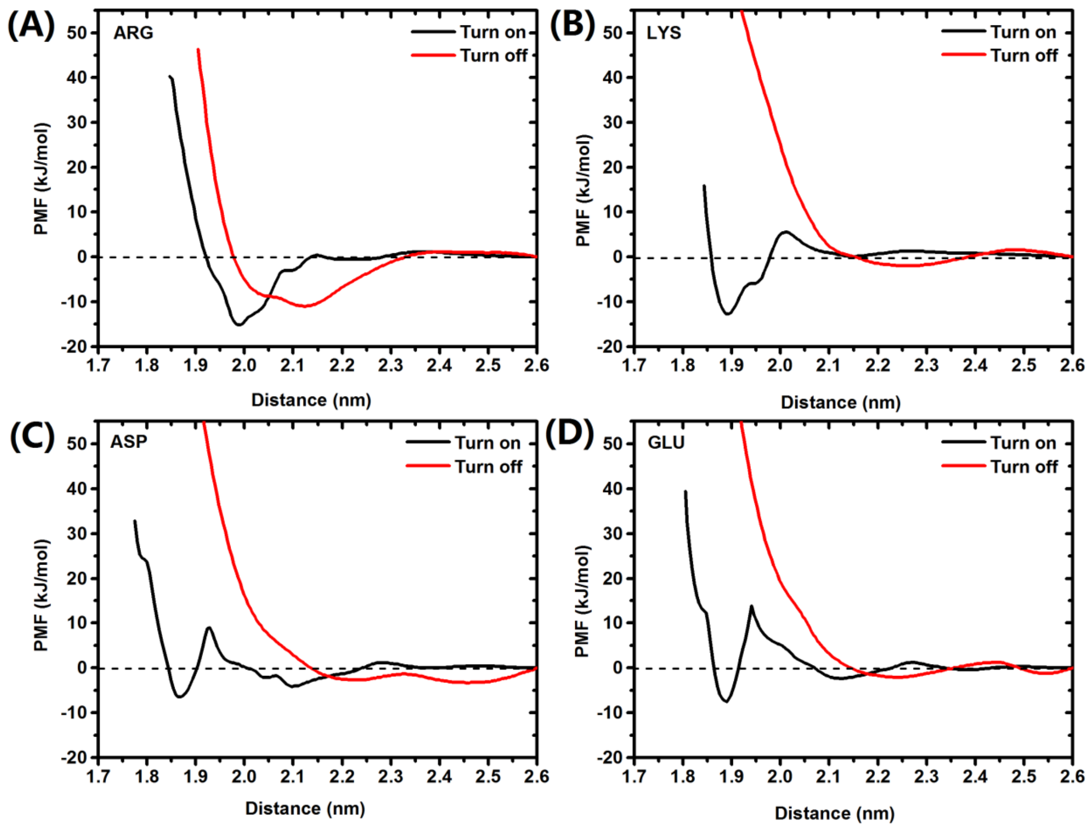

Fig. S4. Free energy profile of charged amino acids in the states of turn-on and turn-off TiO_2 NP atomic partial charge.

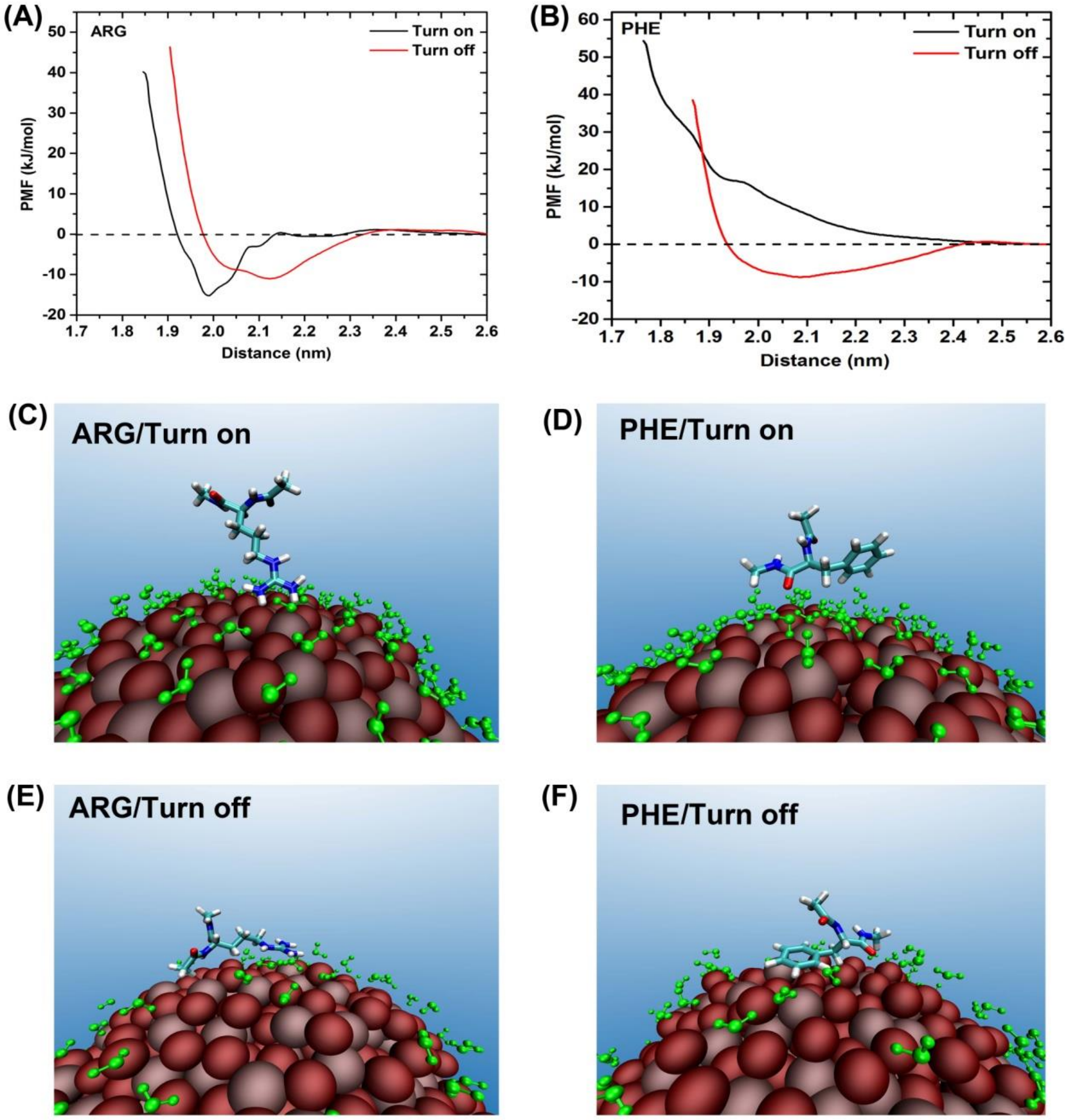

Fig. S5. Free energy profile of Arg and Phe when turning off TiO2 NP atomic charge (**A-B**), and their representative configurations (**C-F**).
